# Supplementary material for: Causal Relationship between Aging and Anorexia Nervosa: A White-Matter-Microstructure-Mediated Mendelian Randomization Analysis
Source: Biomedicines. 2024 Aug 16;12(8):1874. doi: 10.3390/biomedicines12081874 (PMC11351342; doi:10.3390/biomedicines12081874)
Supplement: Supplementary file 1 [file biomedicines-12-01874-s001.zip › Supplementary Figure/Figure S8/Leaveoneout plot of MR analysis for TL to ubm-b-1540.pdf]

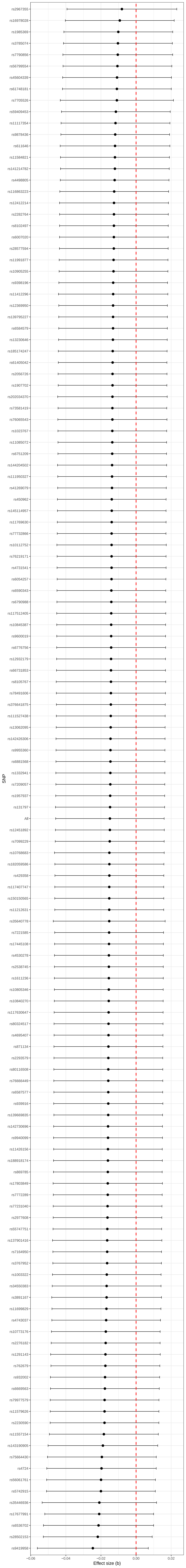

rs2967355
rs16978028
rs1985369
rs3785074
rs7790856
rs56799554
rs45604339
rs61748181
rs7705526
rs59409453
rs11117354
rs9878436
rs611646
rs11584821
rs141214782
rs4498805
rs116863223
rs12412214
rs2282764
rs8102497
rs6007020
rs28577594
rs11991877
rs10905255
rs9398196
rs11412296
rs12369950
rs139795227
rs6584579
rs13230646
rs185174247
rs61405042
rs2056726
rs1907702
rs202034370
rs73581419
rs76065543
rs1023767
rs11085072
rs6751209
rs144204502
rs111950327
rs41269079
rs450962
rs145114957
rs11769630
rs77732866
rs10112752
rs76219171
rs4731541
rs6054257
rs6590343
rs6790988
rs117512405
rs10845387
rs9600019
rs6776756
rs12932179
rs66731853
rs8105767
rs78491606
rs376641875
rs111527438
rs13062095
rs142426306
rs9955360
rs6881568
rs1332941
rs7209057
rs1957937
rs131797
All
rs12451892
rs7099229
rs10768683
rs182059586
rs429358
rs117407747
rs150150565
rs11212631
rs35640778
rs7221585
rs17445108
rs4530278
rs2538745
rs1611236
rs10805346
rs10840270
rs117630647
rs80324517
rs4695407
rs871134
rs2293579
rs80116508
rs76666449
rs6587577
rs939916
rs139669835
rs142730696
rs9940099
rs11426156
rs188918174
rs869785
rs17803849
rs7772289
rs77231040
rs2977608
rs55747751
rs137901416
rs7164950
rs3767952
rs1003322
rs34550383
rs3891167
rs11699829
rs4743037
rs10773176
rs2276182
rs1291143
rs762679
rs932002
rs6669563
rs79977579
rs11579626
rs2230590
rs11557154
rs143190905
rs75664430
rs4724
rs56061761
rs5742915
rs35446936
rs17677991
rs6536702
rs28502153
rs9419958
SNP
−0.06
−0.04
−0.02
0.00
0.02
Effect size (b)
